# Laparoscopic approach for irreducible hernia: A consideration

**DOI:** 10.4103/0972-9941.58506

**Published:** 2009

**Authors:** Viroj Wiwanitkit

**Affiliations:** Department of Surgery, Wiwanitkit House, Bangkhae, Bangkok 10160, Thailand

Dear Editor,

I read the current publication of Jagad *et al*. with great interest.[1] Jagad *et al*., reported their experience and success on using the laparoscopic transperitoneal approach for irreducible inguinal hernias.[1] I agree with this minimally invasive surgical approach. However, the point to be addressed is the importance of case selection and patient preparation to exclude the clinically poor and risk case that needs a standard classical approach. Nevertheless, the data on the comparative cost-effectiveness and cost utility (which should also focus on the hospitalization and pharmacological need) should be clarified. These data help assure us to shift to a new approach for irreducible hernia.
